# Vitamin D and inflammatory markers: cross-sectional analyses using data from the English Longitudinal Study of Ageing (ELSA)

**DOI:** 10.1017/jns.2016.37

**Published:** 2017-01-12

**Authors:** Cesar de Oliveira, Jane P. Biddulph, Vasant Hirani, Ione Jayce Ceola Schneider

**Affiliations:** 1 Department of Epidemiology & Public Health, English Longitudinal Study of Ageing, University College London, London, UK; 2 Centre for Education and Research on Ageing, School of Public Health, ARC Centre of Excellence in Population Ageing Research, University of Sydney, Sydney, New South Wales, Australia; 3 Universidade Federal de Santa Catarina, Campus Araranguá, Santa Catarina, Brazil; 4 Department of Epidemiology & Public Health, University College London, London, UK

**Keywords:** Vitamin D, Inflammation, Older adults, Ageing, Prospective studies, 25(OH)D, 25-hydroxyvitamin D, CRP, C-reactive protein, ELSA, English Longitudinal Study of Ageing, WBC, white blood cell count

## Abstract

Recent evidence suggests that low vitamin D concentrations are associated with increased levels of inflammatory markers. However, there are limited studies investigating associations between vitamin D levels and inflammatory markers in the general population and much of this evidence in older adults is inconclusive. Therefore, this study investigates the cross-sectional association of serum 25-hydroxyvitamin D (25(OH)D) levels with inflammatory markers in 5870 older English adults from wave 6 (2012–2013) of the English Longitudinal Study of Ageing (ELSA). ELSA is a large prospective observational study of community-dwelling people aged 50 years and over in England. Serum 25(OH)D levels, C-reactive protein (CRP) levels, plasma fibrinogen levels, white blood cell count (WBC), age, season of blood collection, waist circumference, total non-pension household wealth, measures of health and health behaviours that included depression, number of cardiovascular, non-cardiovascular conditions and difficulties in activities of daily living, smoking, and physical activity were measured. There was a significant negative association between low 25(OH)D levels (≤30 nmol/l) and CRP (OR 1·23, 95 % CI 1·00, 1·51) and WBC (OR 1·35, 95 % CI 1·13, 1·60) that remained after adjustment for a wide range of covariates of clinical significance. However, for fibrinogen, the association did not remain significant when waist circumference was entered in the final model. Our findings showed that 25(OH)D levels were associated with two out the three inflammatory markers investigated. The independent and inverse association between serum 25(OH)D levels and inflammation suggests a potential anti-inflammatory role for vitamin D in older English individuals from the general population.

Low vitamin D status is an increasingly important public health issue worldwide, in all population groups, although more common in older adults^(^^1^^)^. The serum concentrations of 25-hydroxyvitamin D (25(OH)D), the major storage and circulating form of vitamin D, rise and fall with the supply of vitamin D_3_ (cholecalciferol) and vitamin D_2_ (ergocalciferol). Vitamin D_3_ is metabolised to 25(OH)D_3_ in the liver by vitamin D 25-hydroxylase and then further hydroxylated by the key enzyme 25-hydroxylvitamin D_3_-1α-hydroxylase (CYP27B1) to the biologically active form: 1,25(OH)D or calcitriol in the kidney and in many different tissues throughout the body^(^^2^^)^. Older adults are at increased risk of poor vitamin D status due to the lack of sun exposure and to an age-related decline in the efficiency of vitamin D synthesis and metabolism^(^^3^^)^.

The importance of vitamin D in the absorption and metabolism of Ca for bone health is well known^(^^2^^)^. Further studies have demonstrated that low 25(OH)D concentrations may promote the pathogenesis of type 1 diabetes^(^^3^^)^, rheumatoid arthritis^(^^4^^)^, multiple sclerosis^(^^5^^)^, cancer^(^^6^^)^, sarcopenia^(^^7^^)^ and other diseases. Other actions of vitamin D include its impact on innate and adaptive immunity^(^^8^^)^. High concentrations of inflammatory biomarkers, such as plasma fibrinogen, white blood cell count (WBC) or C-reactive protein (CRP), have been associated with chronic inflammatory diseases, such as cardiometabolic disease risk^(^^9^^)^. Some studies have demonstrated that high 25(OH)D concentrations may protect against CVD^(^^10^^–^^12^^)^.

It is now well recognised that CYP27B1 and the vitamin D receptor are expressed in cells involved in the inflammation/immune system in the human body^(^^1^,^3^^)^. This provides biologically plausible reasons for why low vitamin D levels could play a role in the aetiology of inflammatory diseases such as cardiometabolic diseases but the majority showing a link are based on small samples or on specific patient groups^(^^14^^,^^15^^)^.

There are limited studies investigating the associations between 25(OH)D serum levels and inflammatory markers in the general population^(^^16^^–^^18^^)^ and much of the evidence in older adults is inconclusive. Therefore, the aim of this cross-sectional analysis was to investigate the associations between the 25(OH)D concentrations and three markers of inflammation (CRP, fibrinogen and WBC) using data from the English Longitudinal Study of Ageing (ELSA). ELSA was designed to be nationally representative of community-dwelling adults aged 50 years and over in the general population of England, UK.

## Methods

### Study population

ELSA is an ongoing prospective observational study of community-dwelling people aged 50 years and over in England that commenced in 2002. The ELSA sample was drawn from participants who had previously participated in the Health Survey for England; an annual health examination survey, which each year recruits a different nationally representative sample using a multi-staged stratified random probability design^(^^19^^)^. After baseline, follow-up interviews within ELSA occur every 2 years and health examinations, i.e. a nurse visit, every 4 years. The first health examination was in 2004–2005. A detailed description of the study can be found elsewhere^(^^20^^)^. Analyses for this study used cross-sectional data from wave 6 (2012–2013) as this was the first time that 25(OH)D concentrations were ascertained in ELSA. From wave 6, 5870 participants had 25(OH)D data.

### Assessment of 25-hydroxyvitamin D

Wave 6 had 10 601 respondents that also included non-core members, such as partners. Only the 9169 core sample members eligible for a nurse visit at which blood samples could be taken were included. Of those, 7730 had a nurse visit. Blood samples were obtained from 6206 participants, and 25(OH)D concentrations were ascertained in 5870. Blood samples were not taken from those who had a clotting or bleeding disorder (e.g. haemophilia or low platelets), had ever had a fit, were currently on anticoagulant drugs (e.g. warfarin therapy) or did not give their consent in writing. The analyses of blood samples were carried out at the Royal Victoria Infirmary (Newcastle upon Tyne, UK). Serum 25(OH)D levels were measured by the Diasorin Liaison immunoassay that detects both 25(OH)D_2_ and 25(OH)D_3_ and therefore provides the total circulating 25(OH)D level, as previously described^(^^21^^)^. The assay for 25(OH)D has an analytical sensitivity (lower detection limit) of 7·5 nmol/l. The detection limit represents the lowest measurable analyte level that can be distinguished from zero. All assays were performed in duplicate. The CV ranged from 8·7 to 9·4 %. The laboratory performing the 25(OH)D analyses took part in the Internal and the Vitamin D External Quality Assessment Schemes (DEQAS).

### Inflammatory markers

Three inflammatory markers were measured at wave 6 (2012–2013): CRP, plasma fibrinogen and WBC. Further details of the blood sample analyses, the internal quality control, and the external quality assessment of the laboratory can be obtained from the 2004 Health Survey for England technical report^(^^22^^)^ since both the Health Survey for England and ELSA employed the same laboratory and the same guidelines and protocols for the blood analyses.

### Covariates

Total non-pension household wealth included financial wealth (savings and investments), the value of any home and other property (less mortgage), the value of any business assets and physical wealth such as artwork and jewellery, net of debt. Wealth is the most robust indicator of socio-economic circumstances in ELSA, and has been found to be more strongly associated with the risk of death than any other socio-economic position indicator at older ages^(^^23^^)^. The number of co-morbidities, i.e. CVD and other chronic conditions, was assessed by self-reported doctor-diagnosed chronic diseases that included diabetes, cancer, stroke, arthritis, lung disease, and Parkinson's and for CVD included high blood pressure, angina, heart attack, heart failure, heart murmur or heart rhythm. Smoking status was classified into non-smokers, former smokers or current smokers. Self-reported physical activity included questions about the frequency of participation in vigorous, moderate, mild and sedentary physical activities: more than once per week, once per week, one to three times per month, hardly ever. Depressive symptoms were measured by the shortened version of the Center for Epidemiological Studies-Depression (CES-D) scale^(^^24^^,^^25^^)^. A dichotomous variable for depression was derived using the validated cut point of four or more depressive symptoms to classify depression^(^^25^^)^. Physical functioning was measured using self-reported limitations in the following six basic activities of daily living: dressing, walking across a room, bathing or showering, eating, getting in or out of bed, using the toilet. A physical functioning limitation was defined as having a limitation in one or more activities. Waist circumference was categorised into three main groups using sex-specific cut-offs: low (<94 cm for men and <80 cm for women), medium (≥94 cm and <102 cm for men; ≥80 cm and <88 cm for women) and high (≥102 cm for men and ≥88 cm for women).

### Statistical analyses

Logistic regression was used to investigate the unadjusted and adjusted association between 25(OH)D levels with each of the three inflammatory markers. Normal levels for fibrinogen (≤3·8 g/l)^(^^26^^)^ and CRP (<3 mg/l)^(^^27^^)^ and below the median for WBC (6·3 × 10^9^ cells/l) were used as reference categories. 25(OH)D levels, the independent variable, was categorised into quartiles: the lowest quartile (≤30 nmol/l), second quartile (30·01 until 46·00 nmol/l), third quartile (46·01 until 64·00 nmol/l) and the highest quartile (>64·01 nmol/l; reference category). Following the unadjusted model (model 1), a further four models (models 2 to 5) were derived to investigate the association between 25(OH)D with each outcome variable (fibrinogen, CRP and WBC) and adjusted for potential confounders. Each subsequent model included the variables that were included in the previous model. Model 2 adjusted for demographic and socio-economic factors (age, sex and wealth) and the season of the blood sampling. Model 3 further adjusted for health behaviours; smoking and physical activity, model 4 adjusted model 3 further for health; depression, number of cardiovascular conditions, number of non-cardiovascular chronic conditions and difficulties in activities of daily living, and in the final model 5, model 4 was further adjusted for waist circumference. The analyses were performed using STATA 13.0 (StataCorp LP).

### Ethics approval and informed consent

All participants gave written informed consent. The National Research Ethics Service (London Multicentre Research Ethics Committee (MREC/01/2/91) has approved the ELSA.

## Results

Of the 9169 core participants of wave 6, 25(OH)D levels in 5870 participants were ascertained. Those for whom 25(OH)D data were available (*n* 5870) compared with those for whom data were not available (*n* 3299) were younger; mean age of 66·9 *v.* 69·4 years, with a lower proportion of females; 55·0 % *v.* 56·6 %, and were wealthier; 14·9 % in the lowest quintile of wealth *v.* 21·1 %. Table 1 summarises the characteristics of the sample for which 25(OH)D levels were ascertained. Of the blood samples, 42 % were taken in autumn. Of the subjects, 12 % reported depressive symptoms, and 61 and 56 % reported cardiovascular and non-cardiovascular chronic conditions, respectively. The majority (85 %) did not report difficulties in performing activities of daily living. 25(OH)D levels ranged between 9 and 239 nmol/l and the mean was 48·70 (sd 23·57) nmol/l. CRP levels ranged from 0·10 to 9·80 mg/l, the mean was 2·13 (sd 1·93) mg/l and 23·5 % of the sample had high levels. Plasma fibrinogen levels ranged from 1·20 to 6·30 g/l, the mean was 2·97 (sd 0·54) g/l and 8·0 % of the respondents had levels higher than the normal. WBC ranged from 1·22 to 32·32 (×10^9^ cells/l), the mean was 6·51 (sd 1·96) ×10^9^ cells/l and the median 6·30 ×10^9^ cells/l.

**Table 1.** Characteristics of the analytical sample of men and women aged 50 years and older from the English Longitudinal Study of Ageing (2012–2013) and who had a measure of serum 25-hydroxyvitamin D (Numbers of subjects and percentages)

Variable	*n*	%
Sex (*n* 5870)		
Male	2640	45·0
Female	3230	55·0
Age group (years) (*n* 5870)		
50–59	1369	23·2
60–69	2393	40·8
70–79	1542	26·3
80+	566	9·6
Wealth (*n* 5752)		
Lowest quintile	854	14·9
2nd quintile	1079	18·8
3rd quintile	1227	21·3
4th quintile	1274	22·2
Highest quintile	1318	22·9
Season (*n* 5870)		
Winter	1560	26·6
Spring	437	7·4
Summer	1387	23·6
Autumn	2486	42·4
Smoking status (*n* 5870)		
Non-smoker	2241	38·2
Former smoker	2963	50·5
Current smoker	666	11·4
Physical activity (*n* 5870)		
Sedentary	274	4·7
Mild	870	14·8
Moderate	2828	48·2
High	1898	32·3
Depressive status (*n* 5819)		
No	5119	88·0
Yes	700	12·0
Number of cardiovascular conditions (*n* 5870)		
None	2264	38·6
1	1771	30·2
2+	1835	31·3
Number of non-cardiovascular chronic conditions (*n* 5870)		
None	2562	43·7
1	2200	37·5
2+	1108	18·9
Difficulties in activities of daily living (*n* 5870)		
None	4960	84·5
1+	910	15·5
Waist circumference (*n* 5771)		
Low	1344	23·3
Medium	1477	25·6
High	2950	51·1

In the unadjusted analyses, all three inflammatory markers were associated with 25(OH)D, with low levels of 25(OH)D being associated with higher levels of CRP, fibrinogen and WBC (Table 2). Both the lowest and the second-lowest quartiles of 25(OH)D were significantly associated with higher levels of CRP in the unadjusted analysis (OR 1·69, 95 % CI 1·41, 2·02, *P* < 0·001; OR 1·34, 95 % CI 1·12, 1·61, *P* = 0·002; Table 2, model 1). This association remained statistically significant only for those in the lowest quartile even after the adjustment for all covariates included in the models but with attenuation in the OR from model 2 to model 5 (Table 2). For WBC, respondents in the lowest (OR 1·73, 95 % CI 1·49, 2·01, *P*<0·001) and second-lowest (OR 1·26, 95 % CI 1·09, 1·47, *P* = 0·002) quartiles of 25(OH)D had a greater likelihood of having higher levels of white blood cells than those in the highest quartile (Table 2, model 1). This association remained statistically significant even after adjustment for all covariates included in the models but with a decline in OR from model 2 to model 5 (Table 2). Regarding fibrinogen, only the lowest quartile of 25(OH)D was significantly associated with higher levels of fibrinogen in the unadjusted analysis (OR 1·80, 95 % CI 1·38, 2·35, *P* < 0·001; Table 2, model 1). This association remained statistically significant only for those in the lowest quartile but with a decline in OR from model 2 to model 4. However, this association did not remain significant with adjustment by waist circumference (model 5, Table 2), albeit that the effect estimate was of similar magnitude to the final model with an outcome of CRP.

**Table 2.** Unadjusted and multivariable logistic regression investigating the association between 25-hydroxyvitamin D (25(OH)D) concentrations with inflammatory markers† (Odds ratios and 95 % confidence intervals)

	Model 1 (unadjusted)		Model 2		Model 3		Model 4		Model 5	
	OR	95 % CI	OR	95 % CI	OR	95 % CI	OR	95 % CI	OR	95 % CI
C-reactive protein										
25(OH)D										
Highest quartile	1	–	1	–	1	–	1	–	1	–
3rd quartile	1·17	0·97, 1·40	1·16	0·96, 1·40	1·15	0·95, 1·39	1·16	0·96, 1·40	1·06	0·87, 1·29
2nd quartile	1·34*	1·12, 1·61	1·32*	1·10, 1·60	1·28*	1·06, 1·55	1·27*	1·04, 1·53	1·11	0·91, 1·35
Lowest quartile	1·69*	1·41, 2·02	1·59*	1·31, 1·93	1·42*	1·17, 1·73	1·43*	1·17, 1·75	1·23*	1·00, 1·51
Fibrinogen										
25(OH)D										
Highest quartile	1	–	1	–	1	–	1	–	1	–
3rd quartile	1·09	0·81, 1·46	1·06	0·79, 1·43	1·06	0·79, 1·42	1·05	0·78, 1·41	0·98	0·73, 1·33
2nd quartile	1·04	0·77, 1·40	1·03	0·76, 1·39	0·98	0·72, 1·33	0·95	0·70, 1·29	0·85	0·62, 1·16
Lowest quartile	1·80*	1·38, 2·35	1·66*	1·25, 2·22	1·40*	1·04, 1·87	1·37*	1·02, 1·84	1·22	0·90, 1·66
White blood cell count										
25(OH)D										
3rd quartile	1·17*	1·01, 1·35	1·13	0·98, 1·32	1·12	0·96, 1·31	1·14	0·98, 1·33	1·09	0·93, 1·28
2nd quartile	1·26*	1·09, 1·47	1·26*	1·08, 1·46	1·18*	1·01, 1·39	1·19*	1·01, 1·40	1·11	0·94, 1·31
Lowest quartile	1·73*	1·49, 2·01	1·70*	1·45, 2·00	1·44*	1·22, 1·72	1·45*	1·22, 1·72	1·35*	1·13, 1·60

* *P* < 0.001.

† Model 1: 25(OH)D (unadjusted). Model 2: model 1 + adjusted for sex, age group, wealth, and season. Model 3: model 2 + adjusted for smoking, and physical exercise. Model 4: model 3 + adjusted for depression, number of cardiovascular conditions, number of non-cardiovascular conditions, and difficulties in activities of daily living. Model 5: model 4 + adjusted for waist circumference.

## Discussion

In this large, nationally representative, sample of adults aged 50 years and over, we have demonstrated that older English adults from the general population who have low serum levels of 25(OH)D have increased inflammatory biomarker profiles, including increased CRP, plasma fibrinogen and WBC. This study observed significant negative associations between 25(OH)D and the inflammatory markers CRP and WBC. For fibrinogen, the association did not remain significant in the fully adjusted model when waist circumference was included in the final model. Our findings suggest that insufficient 25(OH)D levels may have the potential to affect the inflammatory response, particularly within the older adult population.

Little research has investigated associations between low 25(OH)D levels and inflammatory markers in the general population, especially in apparently healthy older adults. Most of the previous studies have been conducted in young adults with chronic inflammatory conditions, and controversy remains about the optimal serum 25(OH)D levels for health in older age. In addition, the results from studies investigating the associations between 25(OH)D concentrations with CRP or fibrinogen concentrations are conflicting and studies on the association between 25(OH)D concentrations and WBC are rare^(^^28^^)^. Nevertheless, we cannot ignore that low 25(OH)D levels could represent a marker of ill health resulting from inflammatory processes, a hypothesis that has been raised from a very recent systematic review documenting a discrepancy between the existing observational and intervention studies on the role of 25(OH)D concentrations in non-skeletal health outcomes^(^^29^^)^.

In the present study, CRP levels showed significant negative associations with 25(OH)D levels. Limited data from observational studies^(^^18^^,^^30^^–^^35^^)^ lend support to a primary anti-inflammatory role of vitamin D. In a very recent observational investigation conducted in 957 older Irish adults (>60 years of age), Laird *et al*.^(^^32^^)^ showed a significant association between low vitamin D status (25(OH)D < 25 nmol/l) and markers of inflammation including IL-6, TNF-α, IL-10, CRP and the ratio of IL-6 to IL-10. Yet, not all epidemiological studies reported linear inverse associations between the measures. Shea *et al*.^(^^34^^)^ studied the relationship of vitamin D with several inflammatory markers cross-sectionally in 1381 subjects from the Framingham Offspring Study cohort and did not find a significant association for most of the markers, including CRP. Another, smaller study by Michos *et al*.^(^^17^^)^ did also not find a significant association between vitamin D and CRP.

Evidence from an interventional study investigating the effect of vitamin D supplementation on selected inflammatory biomarkers in older adults using secondary data from a randomised, placebo-controlled trial found little evidence of an effect of vitamin D supplementation on cytokine or adipokine levels, with the possible exception of IL-6^(^^35^^)^. A recent study^(^^36^^)^, investigating the association between serum 25(OH)D and CRP and a potential causal effect by using genetic variants in bi-directional Mendelian randomisation analysis, in the Rotterdam Study, a prospective population-based cohort, showed that serum vitamin D was inversely associated with CRP. However, the results from the Mendelian randomisation analyses did not provide evidence for a causal association.

A possible reason for the lack of correlation between 25(OH)D and CRP in some studies was that they contained younger participants that may have diluted the effect estimate while older participants were not investigated separately. Indeed, ageing itself is associated with low-grade chronic inflammation and elevated CRP and in light of the aforementioned associations with 25(OH)D status, a stronger effect of 25(OH)D may thus be more distinctly observed in the elderly compared with younger individuals in whom CRP levels are expectedly lower^(^^37^^)^.

Our findings showed that fibrinogen did not maintain its negative association with 25(OH)D levels when waist circumference was included in the final model. Similarly, a large study that included 6538 individuals from the 1958 British Birth Cohort^(^^38^^)^ found an inverse association between 25(OH)D and fibrinogen in analyses adjusted for sex and month of blood draw. Yet, these associations vanished after additional adjustment for obesity, lifestyle and social characteristics. As with CRP, previous studies revealed conflicting results regarding the association between 25(OH)D and fibrinogen^(^^39^^,^^40^^)^. The conflicting results are probably due to differences in study design and the selection of specific patient groups. In contrast to the studies that reported no association between the measures, several studies reported an inverse relationship between 25(OH)D and fibrinogen concentrations^(^^28^^)^.

Studies investigating the relationship between 25(OH)D and WBC are sparse. WBC in the present analyses showed a significant inverse association with 25(OH)D. Similarly, one recent study^(^^28^^)^ examining the associations of 25(OH)D with high-sensitivity CRP, fibrinogen and WBC in 2723 men and women aged 25 to 88 years from the first follow-up of the Study of Health in Pomerania also confirmed a potential role of 25(OH)D in chronic inflammation. The authors observed beneficial effects of increasing 25(OH)D for fibrinogen and WBC (in smokers only). On the other hand, a study assessing the relationship between 25(OH)D and WBC in a large hospital population including 1557 adult subjects without chronic kidney disease and 340 adult patients with chronic kidney disease showed no association between the two measures, in adults with or without chronic kidney disease^(^^41^^)^.

Our study has several strengths and potential limitations that need to be considered. A major strength is the large and representative sample of community-dwelling English men and women aged 50 years and older. In addition, certified examiners following standardised protocols, assuring excellent quality of data, performed all examinations and laboratory measurements. Limitations arise from the cross-sectional study design, which investigates associations but cannot provide evidence of causality. However, ELSA is planning to measure levels of vitamin D again in its eighth wave (2016–2017) which will enable longitudinal analyses utilising these repeated measures. Our analyses are based on single-occasion biomarker measurements, which might not adequately represent the participants' inflammatory status. Additionally, we were unable to assess differences between vitamin D_2_ and vitamin D_3_, as the respective data were not available in ELSA. The status on treatment with anti-inflammatory drugs was not documented in our study group. However, even without taking this into account, in our cohort we show an inverse correlation between CRP and 25(OH)D in different groups that is consistent and in agreement with other reports, which similarly also did not correct for this variable. Finally, it was not possible to explore the effect of ethnicity in our analysis, which might act as a confounder due to potentially different rates of inflammatory disease in different ethnic groups, since 98 % of ELSA participants are white English.

In summary, our study suggests a potential role of 25(OH)D in chronic inflammation. Our findings, therefore, contribute significantly to the body of evidence supporting a role for vitamin D in inflammatory conditions. Controversy exists as to whether vitamin D lowers inflammation or whether inflammation lowers 25(OH)D concentrations. Whether our findings have any clinical meaning needs to be further evaluated in randomised controlled clinical trials, especially the potential effects of vitamin D supplementation.

## References

[1] Mithal A, Wahl DA, Bonjour JP, (2009) Global vitamin D status and determinants of hypovitaminosis D. Osteoporos Int 20, 1807–1820. 19543765 10.1007/s00198-009-0954-6

[2] Hewison M, Burke F, Evans KN, (2007) Extra-renal 25-hydroxyvitamin D_3_-1α-hydroxylase in human health and disease. J Steroid Biochem Mol Biol 103, 316–321. 17368179 10.1016/j.jsbmb.2006.12.078

[3] Holick MF (2007) Vitamin D deficiency. N Engl J Med 357, 266–281. 17634462 10.1056/NEJMra070553

[4] Hypponen E, Laara E, Reunanen A, (2001) Intake of vitamin D and risk of type 1 diabetes: a birth-cohort study. Lancet 358, 1500–1503. 11705562 10.1016/S0140-6736(01)06580-1

[5] Merlino LA, Curtis J, Mikuls TR, (2004) Vitamin D intake is inversely associated with rheumatoid arthritis – results from the Iowa Women's Health Study. Arthritis Rheum 50, 72–77. 14730601 10.1002/art.11434

[6] Munger KL, Levin LI, Hollis BW, (2006) Serum 25-hydroxyvitamin D levels and risk of multiple sclerosis. JAMA 296, 2832–2838. 17179460 10.1001/jama.296.23.2832

[7] Rondanelli M, Klersy C, Terracol G, (2016) Whey protein, amino acids, and vitamin D supplementation with physical activity increases fat-free mass and strength, functionality, and quality of life and decreases inflammation in sarcopenic elderly. Am J Clin Nutr 103, 830–840. 26864356 10.3945/ajcn.115.113357

[8] Lappe JM, Travers-Gustafson D, Davies KM, (2007) Vitamin D and calcium supplementation reduces cancer risk: results of a randomized trial. Am J Clin Nutr 85, 1586–1591. 17556697 10.1093/ajcn/85.6.1586

[9] Hewison M (2012) Vitamin D and the immune system: new perspectives on an old theme. Rheumc Dis Clin North Am 38, 125–139. 10.1016/j.rdc.2012.03.012 22525848

[10] Chu MP, Alagiakrishnan K & Sadowski C (2010) The cure of ageing: vitamin D – magic or myth? Postgrad Med J 86, 608–616. 20971712 10.1136/pgmj.2010.101121

[11] Lowe GD (2001) The relationship between infection, inflammation, and cardiovascular disease: an overview. Ann Periodontol 6, 1–8. 10.1902/annals.2001.6.1.1 11887452

[12] Elamin MB, Abu Elnour NO, Elamin KB, (2011) Vitamin D and cardiovascular outcomes: a systematic review and meta-analysis. J Clin Endocrinol Metab 96, 1931–1942. 21677037 10.1210/jc.2011-0398

[13] Nagy L, Szanto A, Szatmari I, (2012) Nuclear hormone receptors enable macrophages and dendritic cells to sense their lipid environment and shape their immune response. Physiol Rev 92, 739–789. 22535896 10.1152/physrev.00004.2011

[14] Brennan A, Katz DR, Nunn JD, (1987) Dendritic cells from human tissues express receptors for the immunoregulatory vitamin D_3_ metabolite, dihydroxycholecalciferol. Immunology 61, 457–461. 2832307 PMC1453440

[15] Haque UJ, Bathon JM & Giles JT (2012) Association of vitamin D with cardiometabolic risk factors in rheumatoid arthritis. Arthritis Care Res (Hoboken) 64, 1497–1504. 22555877 10.1002/acr.21715 PMC3462271

[16] Jorgensen SP, Agnholt J, Glerup H, (2010) Clinical trial: vitamin D_3_ treatment in Crohn's disease – a randomized double-blind placebo-controlled study. Aliment Pharmacol Ther 32, 377–383. 20491740 10.1111/j.1365-2036.2010.04355.x

[17] Michos ED, Streeten EA, Ryan KA, (2009) Serum 25-hydroxyvitamin D levels are not associated with subclinical vascular disease or C-reactive protein in the old order Amish. Calcif Tissue Int 84, 195–202. 19148561 10.1007/s00223-008-9209-3 PMC2908302

[18] Ngo DT, Sverdlov AL, McNeil J, (2010) Does vitamin D modulate asymmetric dimethylarginine and C-reactive protein concentrations? Am J Med 123, 335–341. 20362753 10.1016/j.amjmed.2009.09.024

[19] Mindell J, Biddulph JP, Hirani V, (2012) Cohort profile: the Health Survey for England. Int J Epidemiol 41, 1585–1593. 22253315 10.1093/ije/dyr199

[20] Steptoe A, Breeze E, Banks J, (2013) Cohort profile: the English Longitudinal Study of Ageing. Int J Epidemiol 42, 1640–1648. 23143611 10.1093/ije/dys168 PMC3900867

[21] Anonymous (2011) *Vitamin D total (25-hydroxyvitamin D) pack insert*. Roche Diagnostics. 2011-02 V1.

[22] Graig R, Deverill C & Pickering K (2006) Quality control of blood, saliva and urine analytes In Health Survey for England 2004, Methodology and Documentation, vol. 2, pp. 34–41 [K Spronston and J Mindell, editors]. London: The Information Centre.

[23] Demakakos P, Biddulph JP, Bobak M, (2015) Wealth and mortality at older ages: a prospective cohort study. J Epidemiol Community Health 70, 346–353. 26511887 10.1136/jech-2015-206173 PMC4819652

[24] Radloff LS (1977) The CES-D scale: a self-report depression scale for research in the general population. Appl Psych Meas 1, 385–401.

[25] Steffick DE (2000) Documentation of Affective Functioning Measures in the Health and Retirement Study. DR-005. Ann Arbor, MI: HRS Health Working Group.

[26] Linear Chemicals S.L. (2016) Fibrinogen Clauss Method. http://www.linear.es/ficheros/archivos/764_3510301Fibrinogen(35021)ing.pdf (accessed April 2016).

[27] Pearson TA, Mensah GA, Alexander RW, (2003) Markers of inflammation and cardiovascular disease; application to clinical and public health practice: a statement for healthcare professionals from the Centers for Disease Control and Prevention and the American Heart Association. Circulation 107, 499–511. 12551878 10.1161/01.cir.0000052939.59093.45

[28] Mellenthin L, Wallaschofski H, Grotevendt A, (2014) Association between serum vitamin D concentrations and inflammatory markers in the general adult population. Metab Clin Exp 63, 1056–1062. 24928661 10.1016/j.metabol.2014.05.002

[29] Autier P, Boniol M, Pizot C, (2014) Vitamin D status and ill health: a systematic review. Lancet Diabetes Endocrinol 2, 76–89. 24622671 10.1016/S2213-8587(13)70165-7

[30] Amer M & Qayyum R (2012) Relation between serum 25-hydroxyvitamin D and C-reactive protein in asymptomatic adults (from the continuous National Health and Nutrition Examination Survey 2001 to 2006). Am J Cardiol 109, 226–230. 21996139 10.1016/j.amjcard.2011.08.032

[31] Reyman M, Verrijn Stuart AA, van Summeren M, (2013) Vitamin D deficiency in childhood obesity is associated with high levels of circulating inflammatory mediators, and low insulin sensitivity. Int J Obes 38, 46–52. 10.1038/ijo.2013.75 23736361

[32] Laird E, McNulty H, Ward M, (2014) Vitamin D deficiency is associated with inflammation in older Irish adults. J Clin Endocrinol Metab 99, 1807–1815. 24606079 10.1210/jc.2013-3507

[33] Peterson CA & Heffernan ME (2008) Serum tumor necrosis factor-α concentrations are negatively correlated with serum 25(OH)D concentrations in healthy women. J Inflamm (Lond) 5, 10. 18652680 10.1186/1476-9255-5-10 PMC2503979

[34] Shea MK, Booth SL, Massaro JM, (2008) Vitamin K and vitamin D status: associations with inflammatory markers in the Framingham Offspring Study. Am J Epidemiol 167, 313–320. 18006902 10.1093/aje/kwm306 PMC3151653

[35] Waterhouse M, Tran B, Ebeling PR, (2015) Effect of vitamin D supplementation on selected inflammatory biomarkers in older adults: a secondary analysis of data from a randomised, placebo-controlled trial. Br J Nutr 114, 693–699. 26206095 10.1017/S0007114515002366

[36] Liefaard MC, Ligthart S, Vitezova A, (2015) Vitamin D and C-reactive protein: a Mendelian randomization study. PLOS ONE 10, e0131740. 26147588 10.1371/journal.pone.0131740 PMC4492676

[37] Kruit A & Zanen P (2016) The association between vitamin D and C-reactive protein levels in patients with inflammatory and non-inflammatory diseases. Clin Biochem 49, 534–537. 26778547 10.1016/j.clinbiochem.2016.01.002

[38] Hypponen E, Berry D, Cortina-Borja M, (2010) 25-Hydroxyvitamin D and pre-clinical alterations in inflammatory and hemostatic markers: a cross sectional analysis in the 1958 British Birth Cohort. PLoS ONE 5, e10801. 20520739 10.1371/journal.pone.0010801 PMC2875406

[39] Cannell JJ, Grant WB & Holick MF (2014) Vitamin D and inflammation. Dermatoendocrinol 6, e983401. 26413186 10.4161/19381980.2014.983401 PMC4580066

[40] Bjorkman MP, Sorva AJ & Tilvis RS (2009) C-reactive protein and fibrinogen of bedridden older patients in a six-month vitamin D supplementation trial. J Nutr Health Aging 13, 435–439. 19390750 10.1007/s12603-009-0080-3 PMC12880273

[41] Yildirim I, Hur E & Kokturk F (2013) Inflammatory markers: C-reactive protein, erythrocyte sedimentation rate, and leukocyte count in vitamin D deficient patients with and without chronic kidney disease. Int J Endocrinol 2013, 802165. 23878538 10.1155/2013/802165 PMC3710598

